# Utilization and Application of Public Health Data in Descriptive Epidemiology

**DOI:** 10.2188/jea.JE20140182

**Published:** 2014-11-05

**Authors:** Mariko Naito

**Affiliations:** Department of Preventive Medicine, Graduate School of Medicine, Nagoya University, Nagoya, Japan

**Keywords:** utilization, application, public health data, descriptive epidemiology, Japan

Descriptive epidemiology is defined as epidemiological studies and activities with descriptive components that are much stronger than their analytic components or that fall within the descriptive area of the descriptive-analytic spectrum.^1^ Descriptive epidemiology deals with the occurrence of disease, in terms of both geographical comparisons and descriptions of temporal trends.^2^ The methods used to identify the causes of chronic disease have evolved markedly over the past 20 years, particularly in the areas of epidemiological concepts, quantitative and statistical methods, case-control studies, and clinical epidemiology.^3^ Epidemiological methods and biostatistics have especially become increasingly sophisticated. Descriptive studies are positioned at the base of the hierarchy of scientific evidence; nevertheless, their importance as the basic roots of the epidemiologic approach has not changed. In particular, disease prevalence and incidence data perform an essential role in both research and clinical settings.

This issue of *Journal of Epidemiology* includes three descriptive epidemiology studies from Japan regarding prevalence and incidence of disease. Dr. Kabeya and his colleagues present data on the prevalence of diabetes and distribution of HbA1c in Japan.^4^ They estimated the prevalence of diabetes using data from a large-scale cohort study. Registered inhabitants aged 46–75 years from 10 public health center (PHC) areas were included in the initial survey, and those who received annual health checkups in each PHC-administered area were recruited. The age-standardized prevalence of diabetes in 55- to 74-year-old adults was 8.2% in the initial survey in the late 1990s and 10.6% at the 5-year follow-up. These findings suggest that a concerted effort to reduce the number of individuals who progress to diabetes is required to stop the diabetes epidemic.

In another descriptive study, Dr. Doi et al reported an analysis of cross-sectional data on amyotrophic lateral sclerosis (ALS).^5^ Information on ALS patients who received financial aid for treatment of designated intractable diseases was collected from all 47 prefectural offices in Japan. The authors report that the annual crude prevalence and incidence per 100 000 people were 9.9 (95% CI, 9.7–10.1) and 2.2 (95% CI, 2.1–2.3), respectively, in a representative sample of the Japanese population. This is much lower than in the Caucasian populations of Europe and North America.

Dr. Chihara and his colleagues presented data on the incidence of myelodysplastic syndromes (MDS) in Japan.^6^ MDS is a diverse group of clonal hematopoietic stem cell malignancies in the elderly that present with persistent bone marrow failure and peripheral blood cytopenia. The authors analyzed cancer registry data from the Monitoring of Cancer Incidence in Japan project, which was started in 2007 as a national project to pool prefecture-wide cancer registry data throughout Japan using a standardized protocol. The study showed that the age-adjusted incidence of MDS in Japan, standardized to the world population, was 1.6 cases per 100 000 for males and 0.8 cases for females in 2008. These rates are less than half of those in the United States and similar to those in China, though the authors pointed out that the Japanese registry data might have underestimated the incidence. MDS is common in the elderly, who are seldom thoroughly evaluated during diagnosis, so a substantial number of cases may be missed. Accurate evaluation of the health impact of MDS in Japan requires evaluation of disease mortality, continued surveillance, and improvement in the quality of cancer registry data.

It is difficult for a single study to estimate prevalence and incidence in rare diseases. Even for diseases that have high frequency, geographical variation needs to be considered for accurate nation-wide estimates. Utilization and application of public health data for descriptive studies can be useful; however, the use of public health databases for estimating disease prevalence and incidence is a developing field in Japan.

As shown in the study performed by Dr. Chihara,^6^ cancer incidence data in Japan are available from population-based cancer registries (PBCRs). “The Cancer Registry Promotion Act” was enacted on December 6, 2013. This act regulates the collection, processing, release, and use of cancer registry data and clearly addresses the following: (1) cancer reporting will become a legislative duty of hospitals (>20 beds); (2) information collected in each PBCR must be registered in the new database system in the National Cancer Center, Tokyo (NCC); (3) the NCC will follow subjects in the database by linking to national death certificate data files, in order to calculate accurate cancer survival; and (4) the national government will provide financial support to PBCRs to cover part of the cost of registration.^7^ It is expected that this system will promote the conduct of nationwide cohort studies, with cancer incidence, mortality, and survival as the outcome measures.^7^

Furthermore, several national databases collect and store the profiles of health care institutions or individuals who have used health care services, such as the National Database and the Diagnosis Procedure Combination database. Because no database is perfect, linkage across these resources is crucial.^8^ Japan has no unique personal identifiers at present. All Japanese citizens will be issued a national identification number, the so-called My Number, starting in January 2016. While concerns about privacy and security issues may arise, this identification number should be beneficial for medical applications, including research.

High-quality descriptive studies can provide fruitful scientific evidence and have societal relevance. Basic descriptions of the relationships between disease occurrence and the characteristics of person, place, and time remain the fundamental building blocks of epidemiology.^9^ Descriptive analysis is an essential tool in hypothesis development for analytical studies and in monitoring public health policies. However, the basic infrastructure of disease registries and databases is currently insufficient. The disease registry systems and databases for many intractable diseases, such as Behçet’s disease and IgA nephropathy, can be useful for analyzing prognostic factors of these diseases.

Establishment of qualified databases in medical welfare and their mutual linkage plays a considerable role in improving the quality and quantity of research in the big-data era. Skills in data management and analysis are also required. Big data have the potential to revolutionize research,^10^ and this new era also sheds light on the value of descriptive data. The ultimate goal of primary prevention is dependent on the effective interface of descriptive and analytic epidemiology,^9^ and the importance of descriptive epidemiology is growing in Japan.

## References

[1] Porta M. A dictionary of epidemiology fifth edition. New York: Oxford University Press; 2008. p. 64.

[2] Boyle P. Continual importance of descriptive epidemiology in cancer research. Ann Oncol. 1996;7:439–40. 10.1093/oxfordjournals.annonc.a010630 8839896

[3] Savitz AD, Harris RP, Brownson RC. Methods in chronic disease epidemiology. In: Brownson RC, Remington PL, Davis JR, editors. Chronic disease epidemiology and control second edition. Baltimore: United Book Press; 1998. p. 27.

[4] Kabeya Y, Kato M, Isogawa A, Takahashi Y, Matsushita Y, Goto A, . Descriptive epidemiology of diabetes prevalence and HbA1c distributions based on a self-reported questionnaire and a health checkup in the JPHC diabetes study. J Epidemiol. 2014 (in press) doi:10.2188/jea.JE20130196. 10.2188/jea.JE20130196 24998950 PMC4213220

[5] Doi Y, Atsuta N, Sobue G, Morita M, Nakano I; Research Committee of CNS Degenerative Diseases of Japan. Prevalence and incidence of Amyotrophic Lateral Sclerosis in Japan. J Epidemiol. 2014 (in press) doi:10.2188/jea.JE201400509. 10.2188/jea.JE20140059 25373461 PMC4213224

[6] Chihara D, Ito H, Katanoda K, Shibata A, Matsuda T, Sobue T, . Incidence of Myelodysplastic Syndrome in Japan. J Epidemiol. 2014 (in press) doi:10.2188/jea.JE20140042. 10.2188/jea.JE20140042 25088696 PMC4213221

[7] Tanaka H, Matsuda T. Arrival of a new era in Japan with establishment of the Cancer Registration Promotion Act. Eur J Cancer Prev. 2014 (in press). 10.1097/CEJ.0000000000000095 26398323

[8] Ito H. Value of the national databases: It all depends on how we use them. Expert commentaries in National Quality Measures Clearinghouse, Agency for Healthcare Research and Quality, 2011. 10.1080/15360280802537332 21923316

[9] Nasca PC. Descriptive epidemiology and cancer control. N Y State J Med. 1986;86:231–2. 3459077

[10] Weil AR. Big data in health: a new era for research and patient care. Health Aff (Millwood). 2014;33:1110. 10.1377/hlthaff.2014.0689 25006134

